# The prevalence and associated factors of burnout among undergraduates in a university

**DOI:** 10.1097/MD.0000000000026589

**Published:** 2021-07-09

**Authors:** You Li, Liang Cao, Jianyuan Liu, Tai Zhang, Yixing Yang, Wuxiang Shi, Yingjue Wei

**Affiliations:** a Department of Environmental Health and Occupational Medicine, the School of Public Health, Guilin Medical University, Guilin, Guangxi Province, China; b Department of Experimental Teaching Center, the School of Public Health, Guilin Medical University, Guilin, Guangxi Province, China; c Institute of Preventive Medicine, the School of Public Health, Dali University, Dali, Yunnan Province, China; d Health Management Unit, Faculty of Humanities and Management, Guilin Medical University, Guilin, Guangxi Province, China.

**Keywords:** burnout, influencing factors, undergraduates

## Abstract

The study aimed to evaluate the prevalence and possible factors associated with burnout (BO) among undergraduates in a university setting in Dali.

This cross-sectional study involved students enrolled in different specialties. The method of stratified random sampling was used to conduct the investigation. The questionnaire included Maslach Burnout Inventory and the influencing factors. The data were analyzed using SPSS 16.0 (SPSS Inc., Chicago, IL). The quantitative data were compared using *t* tests or ANOVA. Multiple linear regression was used to assess the relationship between BO risk and relevant influencing factors.

The prevalence of study BO was 38.1%. Significant differences of the mean scores on BO and low personal efficacy were observed between men and women, with women obtaining a higher score (*t* = –2.588, *P* = .010; *t* = –2.929, *P* = .003; respectively). The scores of overall BO, emotional exhaustion (EE), and cynicism were low, whereas that of professional efficacy was higher for students with excellent marks (*P* = .000). Students majoring in liberal arts obtained low scores of overall BO, EE, and cynicism. Nevertheless, their score for professional efficacy was higher than those from other specializations (*P* < .05). Total BO, as the dependent variable, revealed that 3 predictors (social factors, school factors, and interpersonal communication) accounted for 30.8% of the variance (*R*^2^ = 0.308). A regression analysis of EE as the dependent variable identified that 2 variables (social and school factors) explained 45.8% of the variance (*R*^2^ = 0.458).

BO among undergraduates is present in a university setting in Dali. A variety of factors including social factors, school factors, and interpersonal communication can influence the prevalence of BO. Therefore, society should strengthen employment and further understand psychology; schools and families must pay attention to the psychological development of college students.

## 1 Introduction

Burnout (BO) is a well-known phenomenon. Burnout syndrome was first pioneered in the 1970s by American psychologist Herbert Freudenberger. It is defined as a state of chronic fatigue, depression, and frustration.^[1,2]^ At the same time, the concept of BO was first applied to the field of psychology.^[3]^ Previous studies examined job BO, which mainly includes teachers, police officers, and medical staffs.^[4–7]^ Research on BO also involves college and middle school students.^[8,9]^ Student BO refers to a psychological syndrome, which occurs in the form of exhaustion from the intense demands of studying. Emotional exhaustion (EE) mainly refers to the inability to deal with problems encountered during the learning process, leading to negative emotions, such as BO and depression. Cynicism (CM) perceived as the development of negative, cynical attitudes from the study and a feeling of low efficacy and academic achievement,^[10]^ such as arriving late, leaving early, skipping classes, and failing to complete academic tasks on time, not necessarily with other people. Low professional efficacy (sense of accomplishment) refers to the low sense of achievement that students feel when completing their school requirements.

Student BO has recently attracted a lot of attention with the large amount of existing literature established on its prevalence around the world. All these occurred because student BO can lead to the following negative aspects. Student BO among college students can affect their physical and mental development, manifesting as drowsiness, fatigue, eating disorder, migraine, emotional instability, depression, myocardial infarction, alcohol use disorder, hypertension, and even illicit drug use.^[11–16]^ Thus, these symptoms can also lead to the waste of educational resource and the decline of the students’ learning ability. Bringing these symptoms into college students’ future jobs may have adverse effects.^[17,18]^

Secondly, individuals’ negative emotions can lead to inattention and student BO. When individuals’ study or work requirements increase, their creativity can decline. Certain student-centered teaching strategies, such as problem-based learning, can also induce high-level stress and distress to students. They doubt the consistency of their training and apprehension during the evaluation process of the content learned. A prevalent feeling among these students is unpreparedness.^[19,20]^ Finally, individuals who receive little support from society, family, and friends tend to experience BO and lead to drop out.^[21]^

Dali City, located in the western frontier minority areas in the Yunnan Province, China, only has few universities. Compared with other universities, the students here have few opportunities to communicate with peers and study in nearby universities, and their lives are not so colorful, which may have impact on the study BO. As student BO is a multi-dimensional phenomenon that is a function of demands on study resources and personal resources, understanding and identifying predictors may allow for early detection and prevention. The study aimed to determine the prevalence and the influencing factors of student BO among undergraduates and to provide a reference to reduce the prevalence of student BO.

## 2 Methods

### 2.1 Participants

The study was a cross-sectional design conducted from the 1st of February to the 30th of March in the School of Public Health, Dali University (Yunnan, China) in 2017. Inclusion criteria included that students from Grade 1 to Grade 4/Grade 5 of Dali University volunteered to participate in our survey. Exclusion criteria included that students wouldn’t participate in the survey. The method of stratified random sampling was used to conduct the investigation. We calculated the sample size according to the following formula. *P* = 30% (referring to the *P* value of other schools, we took .3), α = 0.05 (bilateral), β = 0.1 (unilateral), prediction accuracy (*d*) is 0.1, design effect (*m*) is 2. n = *m* × z^2^_*α/2*_ × *P* × (1 − *P*)/*d*^2^ = 168. Finally, the sample size of each grade was 200 people. One thousand students enrolled in the first to the fourth year level in the university were approached to participate in the survey. Only completed questionnaires—totaling to 860—were included in the analysis. The participants majored in clinical medicine, pharmacy, nursing, preventive medicine, health inspection and quarantine, food quality and safety, Chinese language and literature, art, sports, English, computer, pre-school education, economic management, law, and agriculture. These specializations were divided into 4 fields: medicine, liberal arts, science and engineering, and technical courses. The questionnaire included demographic information (such as age, sex, specialization, scores, and year level). The scores were divided into 4 different segments (excellent, good, passing, and poor grades) according to previous academic records. BO inventory-student survey and the influencing factors of student BO. The study was in accordance with the World Medical Association Declaration of Helsinki. The study was reviewed and approved by the ethics committee of Dali University (The ID number of ethical approvals was 2016–005.). All participants provided written informed consent.

### 2.2 BO measurement

BO was measured using a student BO questionnaire designed by Rong et al.^[22]^ The scale has been widely used among undergraduates in China, and it originally contains 20 items for evaluating BO among undergraduates. In this study, the scale was revised on the basis of Maslach Burnout Inventory-Student Survey^[23]^ so that the measurement is more suitable for Chinese students. The survey consisted of 3 dimensions: EE (8 items), CM (6 items), and low professional efficacy (6 items). Participants who obtained high scores in EE and CM and low scores in academic efficacy were graded with high risk of BO.

Each question used a 5-point Likert scale (1: completely out of line, 2: basically out of line, 3: uncertain, 4: basically in line, 5: fully in line). A reverse scoring method was required if a reverse problem was given. The total score of the scale was 100, and a total score >60 indicated study BO. The higher the score, the more serious the degree of BO. The middle value of each question was 3 points, and we subsequently calculated the average score of each item. The influencing factors of undergraduates’ learning BO were composed of social (3 items), school (5 items), family (3 items), and interpersonal relationships (self-factors, 4 items).

### 2.3 Data analysis

The data were analyzed with SPSS 16.0 (SPSS Inc., Chicago, IL). The quantitative data were compared using *t* tests or ANOVA. Multiple linear regression was used to assess the relationship between BO risk and relevant influencing factors. A *P*-value of <.05 was statistically significant.

## 3 Results

### 3.1 Subject demographics

The demographic characteristics are presented in Table 1. Among the 1000 undergraduate participants, 860 students fully answered the questionnaires. The response rate of the survey was 86%. The sample consisted of 432 male and 428 female students from different year levels (337 freshmen, 280 sophomores, 199 juniors, and 44 seniors). Almost 70% of the students came from rural areas and are of Han nationality. Moreover, 43.6% are majoring in medicine, and most undergraduates (69.3%) have siblings. Participants’ scores accounted for 16.2%, 35.9%, 37.2%, and 0.7% for excellent, good, passing, and poor grades, respectively.

**Table 1:** Sociodemographic characterization of the undergraduates (n = 860).

Variables	N	%
Gender		
Male	432	50.2
Female	428	49.8
Birthplace		
Rural	592	68.8
Urban	268	31.2
Nationality		
Han nationality	606	70.5
Minority	254	29.5
One child		
Yes	264	30.7
No	596	69.3
Grade		
Freshman	337	39.2
Sophomore	280	32.6
Junior	199	23.1
Senior	44	5.1
Type of specialty		
Medicine	375	43.6
Liberal arts	176	20.5
Science	219	25.5
Technical course	90	10.4
Score		
Excellent	139	16.2
Good	309	35.9
Pass	320	37.2
Fail	92	10.7

### 3.2 Prevalence of study BO

The lowest score was 40, with a maximum of 80 for the total scores of study BO. The prevalence of study BO was 38.1% (Table 2).

**Table 2:** The basic situation of study burnout.

Marks	Study burnout	N	%
≤60	No/ Not sure	532	61.9
>60	Yes	328	38.1

### 3.3 Comparison of study BO between men and women

The mean scores on BO and low professional efficacy between men and women were significantly different, with female students obtaining a higher score (*t* = –2.588, *P* = .010; *t* = –2.929, *P* = .003). (Table 3).

**Table 3:** Analysis of study burnout among the undergraduates of different features.

Parameter	n	BO	EE	CM	LPE
Gender					
Male	432	2.91 ± 0.32	2.62 ± 0.79	2.88 ± 0.48	3.31 ± 0.54
Female	428	2.96 ± 0.27	2.64 ± 0.83	2.91 ± 0.51	3.44 ± 0.79
*t*		–2.588	–0.216	–0.716	–2.929
*P*		.010	.829	.474	.003
Grade					
Freshman	337	2.89 ± 0.30	2.47 ± 0.83	2.88 ± 0.51	3.45 ± 0.74
Sophomore	280	2.97 ± 0.28	2.77 ± 0.77	2.90 ± 0.49	3.30 ± 0.65
Junior	199	2.95 ± 0.32	2.71 ± 0.83	2.90 ± 0.50	3.33 ± 0.61
Senior	44	2.95 ± 0.28	2.60 ± 0.70	2.95 ± 0.44	3.41 ± 0.62
*F*		4.092	7.898	0.299	2.932
*P*		.007	≤.001	.826	.033
Score					
Excellent	139	2.84 ± 0.31	2.38 ± 0.86	2.75 ± 0.49	3.54 ± 0.71
Good	309	2.89 ± 0.26	2.51 ± 0.75	2.81 ± 0.46	3.47 ± 0.69
Pass	320	2.98 ± 0.28	2.75 ± 0.73	2.97 ± 0.48	3.28 ± 0.59
Fail	92	3.06 ± 0.39	2.99 ± 0.99	3.12 ± 0.56	3.11 ± 0.76
F		15.847	15.928	16.303	11.854
*P*		≤.001	≤.001	≤.001	≤.001
Birthplace					
Rural	592	2.93 ± 0.30	2.61 ± 0.81	2.89 ± 0.50	3.38 ± 0.68
Urban	268	2.94 ± 0.30	2.67 ± 0.82	2.89 ± 0.48	3.35 ± 0.68
*t*		–0.639	–1.065	0.057	0.715
*P*		.523	.287	.955	.475
Nationality					
Han nationality	607	2.93 ± 0.29	2.62 ± 0.81	2.89 ± 0.49	3.38 ± 0.68
Minority	253	2.94 ± 0.31	2.65 ± 0.81	2.89 ± 0.51	3.36 ± 0.68
*t*		0.362	0.595	–0.013	–0.405
*P*		.718	.552	.989	.685
One child					
Yes	264	2.94 ± 0.31	2.69 ± 0.86	2.91 ± 0.51	3.32 ± 0.72
No	596	2.93 ± 0.29	2.61 ± 0.79	2.89 ± 0.49	3.39 ± 0.66
*t*		0.822	1.379	0.600	–1.428
*P*		.411	.168	.549	.154
Type of specialty					
Liberal arts	176	2.86 ± 0.31	2.35 ± 0.90	2.81 ± 0.56	3.60 ± 0.83
Science	219	2.92 ± 0.29	2.64 ± 0.80	2.89 ± 0.46	3.33 ± 0.64
Technical course	90	3.00 ± 0.27	3.00 ± 0.76	3.00 ± 0.44	2.99 ± 0.68
Medicine	375	2.96 ± 0.30	2.67 ± 0.75	2.91 ± 0.49	3.39 ± 0.57
*F*		5.561	14.043	3.415	17.522
*P*		.001	≤.001	.017	≤.001

BO = burnout, CM = cynicism, EE = emotional exhaustion, LPE = low personal efficacy.

### 3.4 Comparison of study BO among different year levels

Table 3 showed the comparison results among year levels. Freshmen had lower scores of overall BO and EE and higher professional efficacy than other levels (*P* < .05).

### 3.5 Comparison of study BO among different scores

The scores of overall BO, EE, and CM were low, whereas that of low personal efficacy was high for students with excellent marks (*P* ≤ .001) (Table 3).

### 3.6 Comparison of study BO of students from rural and urban areas

No significant differences of BO scores were found between participants who came from rural and urban areas (*P* > .05) (Table 3).

### 3.7 Comparison of study BO between the Han nationals and minorities

The result showed no statistically significant difference in study BO among undergraduates of different ethnicities (*P* > .05) (Table 3).

### 3.8 Comparison of study BO between one child and multiple children

Table 3 indicates no significant difference in study BO between an only child and a child with siblings.

### 3.9 Comparison of study BO between different specializations

Students majoring in the liberal arts obtained low scores of overall BO, EE, and CM. Their score on professional efficacy was higher than that on other specialties (*P* < .05) (Table 3).

### 3.10 Multiple linear regression analysis of the influencing factors of BO

Stepwise multiple regression analysis was conducted to test whether the influencing factors of learning BO (interpersonal, school, family, and social factors) predicted levels of the 4 components of BO (total BO, EE, misconduct, and low sense of achievement). The results are presented in Table 4. Total BO, as the dependent variable, revealed that 3 predictors (social factors, school factors, and interpersonal communication) explained 30.8% of the variance (*R*^2^ = 0.308). The regression analysis of EE as the dependent variable identified that 2 variables (social and school factors) accounted for 45.8% of the variance (*R*^2^ = 0.458). CM, as the dependent variable, indicated that 3 predictors (social factors, school factors, and interpersonal communication) accounted for 31.1% of the variance (*R*^2^ = 0.311). Social, school, and family factors as independent variables interpreted 44.5% (*R*^2^ = 0.445) of personal accomplishment. (Table 4).

**Table 4:** Stepwise multiple regression analysis predicting the average score of burnout.

Variables	*R*	*R*^2^	△*R*^2^	*B*	Sd.	Beta	*t*	*P*
Overall burnout								
Constant				2.262	0.045		49.866	.000
Social factors	0.507	0.258	0.258	0.120	0.013	0.331	8.970	.000
School factors	0.551	0.303	0.045	0.101	0.015	0.260	6.910	.000
Interpersonal communication	0.555	0.308	0.005	0.030	0.0013	0.068	2.312	.021
Emotional exhaustion								
Constant				0.642	0.077		8.317	.000
Social factors	0.621	0.386	0.386	0.395	0.032	0.401	12.289	.000
School factors	0.677	0.458	0.072	0.364	0.034	0.347	10.646	.000
Cynicism								
Constant				1.644	0.075		22.215	.000
Social factors	0.491	0.241	0.241	0.183	0.022	0.303	8.251	.000
School factors	0.540	0.292	0.051	0.164	0.024	0.256	6.821	.000
Interpersonal communication	0.557	0.311	0.019	0.105	0.022	0.143	4.869	.000
Personal accomplishment								
Constant				5.149	0.080		64.281	.000
Social factors	0.602	0.363	0.363	–0.293	0.028	–0.355	–10.572	.000
School factors	0.663	0.439	0.076	–0.290	0.030	–0.330	–9.639	.000
Family factors	0.667	0.445	0.006	–0.087	0.028	–0.089	–3.059	.002

△*R*^2^: Adjusted *R*^2^.

## 4 Discussion

In this study, the prevalence of student BO for undergraduate in Dali was 38.1% according to the appropriate cut-offs in the MBI. In addition, the lowest score was 40, with a maximum of 80 for the total scores of study BO. Our results were similar to the other studies conducted internationally, in which the prevalence of student BO on average was from 15% to 71%.^[24–27]^

We also investigated the relationship between student BO and different demographic characteristics. The results indicated the significant differences of the mean scores on BO and low professional efficacy between men and women, with women receiving a higher score. Our findings are inconsistent with certain literature on sex and BO^[25,28]^ and are related to the professional and geographical inconsistencies among the participants.

In our study, freshmen had lower scores of overall BO and EE and higher low professional efficacy than other levels. These results are consistent with a recent research on BO among medical students at Sun Yat-sen University,^[29]^ suggesting that BO scores are significantly higher for higher year levels. Freshmen mainly learn basic knowledge, which is relatively easy, and they do not have immediate plans for their future career. Thus, they experience lower BO and higher professional efficacy than students from higher year levels.

The scores of all the items on BO were low for the students with excellent marks, except for low professional efficacy. That is, the better the grade, the lower the prevalence of student BO. This result may be related to the effective self-discipline of students and their capacity to learn. Excellent students can plan their future career development.

Our result indicated no significant difference in BO scores between participants who came from rural and urban areas. This finding is inconsistent with the study of the other research, who concluded that rural origin is associated with vulnerability to burn-out in Australian medical students undertaking a rural clinical placement.^[30]^ This may be related to the fact that the participants they selected were medical students who entered rural clinical placement. These factors could include dislocation from home, financial stress, and reduced academic capital in their social networks that contribute to academic stress.

The results confirmed that the current study did not find any significant difference in the relationship between BO and nationality. This result is similar to that of other research.^[31,32]^ With the development of social economy and the continuous optimization of the external environment, differences in living conditions, external environments, and education level among the undergraduates of Han nationality and ethnic minorities lessened.

We found that there wasn’t significant difference in learning BO between being an only child and having siblings. Certain studies reveal that the scores of student BO were significantly higher for an only child than for those with siblings.^[33,34]^ Thus, the current study is inconsistent with existing research because undergraduates receive the same education and face similar challenges regardless of the number of siblings.

Our results indicated that different specializations obtain different scores of BO with low scores of overall BO among those majoring in the liberal arts. Other articles have investigated BO in various fields. The study found that nonclinical workers at a local medical education center experience less BO than the general population.^[35]^ At the same time, a meta-analysis revealed that those specializing in surgery have significantly different rates of BO.^[36]^ The results are completely inconsistent, possibly due to the various demographics in different fields.

BO has multi-factorial origins that are social and personal.^[37]^ Total BO, as the dependent variable, showed that 3 predictors (social factors, school factors, and interpersonal communication) explained 30.8% of the variance (*R*^2^ = 0.308). The regression analysis of EE as the dependent variable identified that 2 variables (social and school factors) accounted for 45.8% of the variance (*R*^2^ = 0.458). CM, as the dependent variable, revealed that 3 predictors (social factors, school factors, and interpersonal communication) accounted for 31.1% of the variance (*R*^2^ = 0.311). Social, school, and family factors as independent variables interpreted 44.5% (*R*^2^ = 0.445) of personal accomplishment.

Social factors, school factors, and interpersonal communication are regarded as the main influencing factors of student BO. Certain studies have explored the association between social support and BO and revealed that social support and self-efficacy are identified as negatively associated with BO.^[38,22]^

The impact of social factors on college students’ study BO mainly comes from 2 aspects.^[38,39]^ One is the severe employment pressure, and the other is the social atmosphere (social support). Colleges and universities generally expand enrollment, causing the number of college graduates to surge each year, and the subsequent employment pressure can aggravate BO. Driven by such negative psychology, college students have become addicted to online games and are indifferent to learning a direct cause of student BO. Social support from teachers, friends, or family members had a better impact on learning BO.^[40]^

School environment also plays an important role in college students’ mood and emotion. The influence is relatively complex. Studying in a conducive learning environment and atmosphere can greatly enhance students’ learning interest and motivation, alleviate learning BO and promote their healthy growth.^[41]^

One of the most important factors in college life is interpersonal communication, including interpersonal relationships with teachers and other students. College students are active in thinking, broad in hobbies, full of energy, and eager for interpersonal communication. They hope to improve their reputation, gain recognition, and secure trust and support from their peers. Most of the people they interact with are of the same age because they leave their parents when they attend college. As a result, they often communicate with roommates, classmates, and fellow villagers. Their interactions also revolve around learning, examinations, entertainment, and emotions. All these interactions have direct effects on their lives and studies. The lack of interpersonal support and trust among students can lead to students’ BO. Consistent with existing literature^[42]^ our findings revealed that family factors have a small influence on the development of BO.

### 4.1 Limitations

This study has several limitations. First, only one university was surveyed so the sample size was limited. Second, the scale was used to investigate the prevalence rate of student BO but the other scales, for example, social support didn’t use. If the analysis was combined with other scales, the results would be richer and more convincing.

## 5 Conclusions

BO among undergraduates is present in a university setting in Dali. A variety of factors can influence the prevalence of BO. Social factors, school factors, and interpersonal communication can affect college students’ BO. Therefore, society should strengthen employment and further understand psychology; schools and families must pay attention to the psychological development of college students.

## Author contributions

**Conceptualization:** You Li, Wuxiang Shi.

**Data curation:** Liang Cao, Jianyuan Liu, Tai Zhang.

**Formal analysis:** Liang Cao.

**Investigation:** Liang Cao, Jianyuan Liu, Tai Zhang.

**Methodology:** Tai Zhang, Yixing Yang.

**Supervision:** Yingjue Wei.

**Validation:** Yingjue Wei.

**Visualization:** Liang Cao.

**Writing – original draft:** You Li.

**Writing – review & editing:** Wuxiang Shi, Yingjue Wei.
